# Supplementary material for: A Likelihood Approach for Real-Time Calibration of Stochastic Compartmental Epidemic Models
Source: PLoS Comput Biol. 2017 Jan 17;13(1):e1005257. doi: 10.1371/journal.pcbi.1005257 (PMC5240920; doi:10.1371/journal.pcbi.1005257)
Supplement: S1 File — (TAR.GZ) [file pcbi.1005257.s014.tar.gz › HSPH_Online-SI-Revision/PF08/n1-mild/n1-mild_PF2_table4.pdf]

1	newly 3 weeks spec	6.	14.58	{0., 31.}	182.	0.582962	newly 3 weeks cumul	40.	51.4	{6., 100.}	75.6	0.399417	newly 5 weeks spec	2.	10.05	{0., 21.}	436.5	0.752013	newly 5 weeks cumul	43.	73.76	{6., 140.}	112.14	0.455338	newly 51 weeks cumul	116.	128.34	{6., 283.}	74.6207	0.49172
2	newly 3 weeks spec	3.	11.5	{2., 24.}	299.333	0.54069	newly 3 weeks cumul	16.	35.42	{9., 69.}	142.	0.345288	newly 5 weeks spec	4.	9.89	{0., 23.}	189.25	0.530952	newly 5 weeks cumul	23.	56.4	{13., 115.}	165.478	0.368424	newly 47 weeks cumul	28.	133.31	{15., 308.}	392.964	0.539318
3	newly 3 weeks spec	13.	18.95	{1., 42.}	100.846	0.505444	newly 3 weeks cumul	35.	63.94	{6., 136.}	121.657	0.435695	newly 5 weeks spec	6.	14.5	{0., 37.}	193.333	0.65766	newly 5 weeks cumul	61.	95.27	{6., 207.}	102.869	0.444066	newly 56 weeks cumul	77.	191.89	{8., 417.}	190.974	0.563461
4	newly 3 weeks spec	9.	13.62	{1., 30.}	97.1111	0.440199	newly 3 weeks cumul	30.	47.15	{10., 89.}	92.1667	0.346923	newly 5 weeks spec	8.	10.4	{0., 23.}	92.25	0.537083	newly 5 weeks cumul	45.	68.58	{12., 138.}	91.7333	0.368116	newly 50 weeks cumul	300.	135.33	{18., 304.}	59.77	0.624767
5	newly 3 weeks spec	10.	9.36	{0., 21.}	79.2	0.672575	newly 3 weeks cumul	42.	34.95	{1., 89.}	68.8333	0.576505	newly 5 weeks spec	5.	7.16	{0., 17.}	119.6	0.688535	newly 5 weeks cumul	52.	50.31	{1., 125.}	76.2115	0.58178	newly 52 weeks cumul	70.	97.21	{1., 223.}	113.643	0.650508
6	newly 3 weeks spec	20.	32.09	{9., 56.}	79.75	0.255013	newly 3 weeks cumul	66.	100.01	{35., 172.}	69.8939	0.224583	newly 5 weeks spec	12.	25.94	{6., 45.}	137.167	0.395763	newly 5 weeks cumul	97.	155.2	{55., 262.}	77.2371	0.241882	newly 56 weeks cumul	201.	347.2	{98., 567.}	94.2786	0.306622
7	newly 3 weeks spec	20.	16.68	{0., 39.}	67.2	0.572055	newly 3 weeks cumul	60.	59.89	{6., 132.}	67.6833	0.447207	newly 5 weeks spec	5.	13.18	{0., 30.}	210.	0.671239	newly 5 weeks cumul	86.	87.76	{7., 199.}	69.7907	0.471501	newly 55 weeks cumul	126.	160.89	{8., 386.}	89.1349	0.520281
8	newly 3 weeks spec	10.	9.28	{0., 21.}	68.	0.528799	newly 3 weeks cumul	44.	32.08	{2., 66.}	53.4091	0.507767	newly 5 weeks spec	12.	6.47	{0., 15.}	59.0833	0.744181	newly 5 weeks cumul	74.	47.46	{3., 93.}	52.9459	0.549299	newly 47 weeks cumul	324.	92.65	{3., 219.}	71.5278	0.928655
9	newly 3 weeks spec	13.	8.28	{0., 17.}	52.	0.526063	newly 3 weeks cumul	57.	28.81	{3., 54.}	52.7193	0.525648	newly 5 weeks spec	5.	6.37	{0., 14.}	97.4	0.584874	newly 5 weeks cumul	72.	42.09	{4., 80.}	50.0417	0.503684	newly 46 weeks cumul	74.	82.1	{4., 176.}	79.1081	0.516301
10	newly 3 weeks spec	5.	9.23	{0., 20.}	141.	0.580873	newly 3 weeks cumul	23.	32.9	{2., 68.}	93.8261	0.468094	newly 5 weeks spec	1.	6.4	{0., 16.}	582.	0.821927	newly 5 weeks cumul	26.	47.06	{2., 96.}	126.	0.507522	newly 47 weeks cumul	26.	85.24	{2., 214.}	265.	0.6317
11	newly 3 weeks spec	6.	7.51	{0., 19.}	109.5	0.744099	newly 3 weeks cumul	21.	25.48	{0., 63.}	99.5238	0.722007	newly 5 weeks spec	11.	5.14	{0., 15.}	71.8182	0.974805	newly 5 weeks cumul	43.	36.95	{0., 93.}	76.3488	0.792054	newly 46 weeks cumul	315.	74.08	{0., 195.}	77.4857	1.32356
12	newly 3 weeks spec	9.	9.64	{0., 21.}	83.3333	0.620658	newly 3 weeks cumul	42.	32.58	{1., 72.}	60.7619	0.60242	newly 5 weeks spec	9.	7.29	{0., 19.}	75.8889	0.797418	newly 5 weeks cumul	57.	48.47	{1., 107.}	65.9123	0.6328	newly 47 weeks cumul	206.	91.21	{1., 213.}	60.0146	0.861033
13	newly 3 weeks spec	6.	4.93	{0., 12.}	84.8333	0.748891	newly 3 weeks cumul	20.	17.52	{0., 40.}	81.7	0.697641	newly 5 weeks spec	0.1	3.69	{0., 10.}	3670.	0.957086	newly 5 weeks cumul	21.	25.35	{0., 59.}	102.143	0.687022	newly 48 weeks cumul	26.	52.57	{0., 134.}	177.731	0.775119
14	newly 3 weeks spec	4.	10.24	{0., 23.}	209.	0.675749	newly 3 weeks cumul	23.	35.69	{1., 89.}	111.261	0.509921	newly 5 weeks spec	3.	6.63	{0., 14.}	188.333	0.70163	newly 5 weeks cumul	29.	50.27	{1., 127.}	128.379	0.544616	newly 52 weeks cumul	29.	89.11	{1., 197.}	253.621	0.658304
15	newly 3 weeks spec	19.	19.41	{4., 39.}	59.8421	0.327909	newly 3 weeks cumul	57.	64.85	{20., 116.}	55.4211	0.229917	newly 5 weeks spec	13.	15.06	{2., 37.}	72.1538	0.363898	newly 5 weeks cumul	84.	96.82	{26., 183.}	57.6429	0.242588	newly 52 weeks cumul	175.	199.96	{32., 483.}	71.2114	0.339329
16	newly 3 weeks spec	24.	13.87	{1., 27.}	50.7083	0.516782	newly 3 weeks cumul	51.	46.29	{8., 83.}	48.6078	0.318258	newly 5 weeks spec	13.	10.22	{0., 23.}	58.9231	0.524873	newly 5 weeks cumul	79.	68.32	{10., 132.}	49.7215	0.350047	newly 50 weeks cumul	230.	131.85	{10., 248.}	53.9783	0.517076
17	newly 3 weeks spec	19.	22.28	{5., 43.}	61.6842	0.292287	newly 3 weeks cumul	42.	72.93	{18., 136.}	96.0714	0.290877	newly 5 weeks spec	16.	16.7	{1., 32.}	60.125	0.395812	newly 5 weeks cumul	69.	109.71	{22., 187.}	86.2174	0.290641	newly 53 weeks cumul	203.	209.54	{29., 381.}	56.335	0.319761
18	newly 3 weeks spec	17.	8.32	{0., 18.}	60.4706	0.768412	newly 3 weeks cumul	55.	30.13	{1., 65.}	55.3636	0.626851	newly 5 weeks spec	17.	6.27	{0., 16.}	65.9412	0.926666	newly 5 weeks cumul	88.	44.26	{1., 97.}	55.5909	0.678212	newly 46 weeks cumul	176.	89.8	{1., 216.}	59.8295	0.793689
19	newly 3 weeks spec	22.	11.96	{0., 26.}	62.1818	0.712682	newly 3 weeks cumul	64.	42.03	{2., 89.}	57.0781	0.528969	newly 5 weeks spec	12.	7.6	{0., 17.}	65.3333	0.726099	newly 5 weeks cumul	88.	58.87	{2., 126.}	57.4659	0.550408	newly 53 weeks cumul	114.	95.92	{2., 224.}	64.9474	0.567024
20	newly 3 weeks spec	10.	13.19	{3., 28.}	74.1	0.310469	newly 3 weeks cumul	38.	45.6	{16., 87.}	57.5789	0.245687	newly 5 weeks spec	11.	10.28	{0., 22.}	63.2727	0.463623	newly 5 weeks cumul	53.	67.27	{23., 129.}	64.3962	0.266816	newly 50 weeks cumul	69.	126.67	{27., 270.}	118.797	0.368381
21	newly 3 weeks spec	11.	10.8	{2., 22.}	57.6364	0.310166	newly 3 weeks cumul	47.	35.61	{14., 55.}	47.9787	0.267469	newly 5 weeks spec	5.	9.42	{1., 21.}	132.	0.458384	newly 5 weeks cumul	63.	55.29	{20., 107.}	51.5079	0.26285	newly 50 weeks cumul	68.	154.78	{24., 438.}	165.559	0.372768
22	newly 3 weeks spec	16.	14.89	{2., 31.}	58.8125	0.383159	newly 3 weeks cumul	38.	52.89	{10., 103.}	76.8684	0.297849	newly 5 weeks spec	16.	11.31	{0., 24.}	58.5625	0.569766	newly 5 weeks cumul	74.	76.44	{14., 151.}	57.6486	0.312763	newly 52 weeks cumul	332.	144.71	{14., 318.}	60.8223	0.602966
23	newly 3 weeks spec	14.	10.59	{0., 22.}	57.5	0.552153	newly 3 weeks cumul	59.	35.52	{3., 69.}	53.6949	0.496623	newly 5 weeks spec	8.	7.62	{0., 18.}	77.5	0.650066	newly 5 weeks cumul	90.	52.45	{3., 103.}	55.5444	0.544805	newly 47 weeks cumul	150.	104.72	{3., 240.}	64.7733	0.628641
24	newly 3 weeks spec	16.	13.31	{1., 29.}	58.9375	0.452409	newly 3 weeks cumul	68.	44.1	{9., 91.}	50.9118	0.423723	newly 5 weeks spec	13.	9.66	{0., 22.}	60.3077	0.58264	newly 5 weeks cumul	98.	64.57	{10., 131.}	51.7245	0.440741	newly 49 weeks cumul	197.	122.91	{12., 275.}	54.4112	0.526659
25	newly 3 weeks spec	13.	6.82	{0., 16.}	68.4615	0.829316	newly 3 weeks cumul	29.	24.53	{1., 63.}	72.4483	0.58398	newly 5 weeks spec	8.	5.16	{0., 14.}	76.	0.867508	newly 5 weeks cumul	44.	36.2	{1., 92.}	72.8636	0.623906	newly 46 weeks cumul	191.	69.03	{1., 183.}	67.4084	0.932073
26	newly 3 weeks spec	5.	12.27	{1., 26.}	177.8	0.51534	newly 3 weeks cumul	24.	40.98	{4., 76.}	104.667	0.373817	newly 5 weeks spec	1.	9.45	{0., 21.}	873.	0.918527	newly 5 weeks cumul	34.	60.69	{7., 113.}	113.265	0.39719	newly 49 weeks cumul	44.	121.78	{12., 282.}	211.091	0.522749
27	newly 3 weeks spec	19.	6.9	{0., 17.}	66.3158	0.9461	newly 3 weeks cumul	39.	24.37	{0., 58.}	60.2821	0.684842	newly 5 weeks spec	10.	5.	{0., 14.}	68.	0.912085	newly 5 weeks cumul	66.	35.08	{0., 81.}	60.5152	0.758849	newly 46 weeks cumul	107.	67.82	{0., 192.}	71.8318	0.851807
28	newly 3 weeks spec	12.	18.44	{0., 43.}	103.833	0.540905	newly 3 weeks cumul	35.	61.29	{5., 119.}	111.057	0.432642	newly 5 weeks spec	8.	14.11	{0., 31.}	131.875	0.642569	newly 5 weeks cumul	50.	91.13	{7., 206.}	120.46	0.470608	newly 52 weeks cumul	232.	188.1	{7., 384.}	58.1207	0.538734
29	newly 3 weeks spec	13.	12.68	{2., 28.}	61.8462	0.393644	newly 3 weeks cumul	47.	41.47	{7., 80.}	51.2553	0.326024	newly 5 weeks spec	5.	10.1	{0., 23.}	147.2	0.546681	newly 5 weeks cumul	61.	61.6	{13., 131.}	58.7869	0.336755	newly 49 weeks cumul	61.	122.86	{15., 270.}	144.393	0.456208
30	newly 3 weeks spec	22.	13.97	{0., 31.}	63.1364	0.633295	newly 3 weeks cumul	51.	46.54	{5., 103.}	64.3137	0.436936	newly 5 weeks spec	11.	10.28	{0., 22.}	78.7273	0.667119	newly 5 weeks cumul	78.	68.21	{5., 145.}	64.9103	0.47148	newly 53 weeks cumul	111.	125.99	{5., 270.}	82.2072	0.504419
31	newly 3 weeks spec	4.	17.7	{6., 34.}	349.	0.59582	newly 3 weeks cumul	23.	60.61	{25., 103.}	168.217	0.384944	newly 5 weeks spec	0.1	13.99	{3., 25.}	13 892.	2.00797	newly 5 weeks cumul	29.	90.29	{33., 155.}	215.69	0.449433	newly 51 weeks cumul	29.	186.31	{47., 379.}	545.207	0.708787
32	newly 3 weeks spec	21.	12.09	{0., 25.}	52.9048	0.599203	newly 3 weeks cumul	44.	42.63	{0., 86.}	57.0227	0.51723	newly 5 weeks spec	15.	9.13	{0., 19.}	55.4	0.595337	newly 5 weeks cumul	72.	62.81	{0., 123.}	53.9861	0.539768	newly 49 weeks cumul	281.	114.79	{0., 229.}	60.4021	0.808718
33	newly 3 weeks spec	13.	13.9	{2., 28.}	60.3077	0.386984	newly 3 weeks cumul	55.	48.14	{9., 87.}	47.3818	0.319144	newly 5 weeks spec	7.	10.6	{0., 23.}	105.429	0.525977	newly 5 weeks cumul	72.	70.55	{12., 131.}	52.9861	0.335774	newly 50 weeks cumul	85.	138.42	{15., 271.}	107.647	0.435885
34	newly 3 weeks spec	12.	12.33	{0., 29.}	71.4167	0.514907	newly 3 weeks cumul	36.	39.92	{3., 85.}	68.5556	0.419459	newly 5 weeks spec	15.	9.01	{0., 23.}	62.6	0.721345	newly 5 weeks cumul	60.	58.75	{3., 128.}	64.7833	0.463399	newly 49 weeks cumul	138.	113.7	{3., 278.}	67.6667	0.591706
35	newly 3 weeks spec	15.	15.26	{3., 30.}	54.5333	0.327844	newly 3 weeks cumul	76.	52.2	{15., 92.}	43.9474	0.333587	newly 5 weeks spec	15.	11.25	{1., 24.}	55.8	0.487578	newly 5 weeks cumul	115.	76.99	{18., 136.}	45.5565	0.362507	newly 51 weeks cumul	153.	149.61	{22., 297.}	59.1569	0.385517
36	newly 3 weeks spec	2.	12.49	{0., 31.}	551.5	0.759302	newly 3 weeks cumul	28.	45.05	{7., 108.}	110.036	0.404234	newly 5 weeks spec	0.1	8.25	{0., 20.}	8186.	1.48991	newly 5 weeks cumul	28.	63.02	{8., 165.}	162.286	0.445743	newly 53 weeks cumul	28.	103.41	{8., 241.}	297.607	0.566105
37	newly 3 weeks spec	11.	14.16	{1., 31.}	78.	0.427	newly 3 weeks cumul	35.	49.99	{9., 106.}	82.2571	0.36231	newly 5 weeks spec	8.	10.99	{0., 23.}	91.625	0.530314	newly 5 weeks cumul	53.	73.61	{12., 158.}	80.1698	0.374736	newly 49 weeks cumul	95.	138.99	{13., 294.}	94.6	0.448367
38	newly 3 weeks spec	9.	12.07	{0., 31.}	101.222	0.602072	newly 3 weeks cumul	41.	43.21	{2., 108.}	77.9268	0.494615	newly 5 weeks spec	5.	7.82	{0., 20.}	124.4	0.630233	newly 5 weeks cumul	58.	60.44	{2., 162.}	78.5517	0.51563	newly 53 weeks cumul	65.	101.59	{2., 241.}	117.769	0.560397
39	newly 3 weeks spec	13.	9.05	{0., 20.}	64.3846	0.659871	newly 3 weeks cumul	39.	30.27	{2., 64.}	60.641	0.51631	newly 5 weeks spec	4.	6.02	{0., 15.}	129.5	0.664637	newly 5 weeks cumul	49.	43.39	{2., 94.}	67.9796	0.531604	newly 51 weeks cumul	78.	75.86	{2., 178.}	83.1026	0.61437
40	newly 3 weeks spec	11.	12.66	{0., 32.}	88.3636	0.631684	newly 3 weeks cumul	36.	46.	{2., 98.}	89.5556	0.531249	newly 5 weeks spec	1.	9.27	{0., 26.}	873.	0.916883	newly 5 weeks cumul	43.	66.23	{2., 161.}	110.767	0.56582	newly 54 weeks cumul	43.	109.12	{2., 275.}	202.465	0.657806
41	newly 3 weeks spec	9.	16.92	{0., 39.}	134.889	0.532353	newly 3 weeks cumul	33.	58.19	{5., 111.}	119.364	0.431965	newly 5 weeks spec	5.	12.45	{0., 26.}	197.	0.63758	newly 5 weeks cumul	42.	84.97	{5., 167.}	144.31	0.47425	newly 55 weeks cumul	48.	151.9	{7., 373.}	255.083	0.587757
42	newly 3 weeks spec	0.1	13.49	{1., 30.}	13 406.	1.8264	newly 3 weeks cumul	0.1	47.86	{7., 98.}	47 766.	2.45637	newly 5 weeks spec	0.1	9.84	{0., 25.}	9768.	1.60913	newly 5 weeks cumul	0.1	69.16	{9., 151.}	69 066.	2.60029	newly 54 weeks cumul	0.1	118.42	{9., 271.}	118 326.	2.79059
43	newly 3 weeks spec	2.	18.11	{1., 37.}	820.5	0.867114	newly 3 weeks cumul	19.	64.48	{9., 121.}	258.842	0.528332	newly 5 weeks spec	2.	11.95	{0., 25.}	523.5	0.771321	newly 5 weeks cumul	24.	90.78	{13., 172.}	296.083	0.553274	newly 56 weeks cumul	27.	150.89	{15., 327.}	475.593	0.680925
44	newly 3 weeks spec	2.	11.29	{1., 24.}	484.5	0.722786	newly 3 weeks cumul	12.	38.93	{8., 76.}	241.917	0.522646	newly 5 weeks spec	0.1	8.06	{0., 19.}	7990.	1.55748	newly 5 weeks cumul	14.	55.61	{9., 114.}	313.929	0.591598	newly 48 weeks cumul	17.	101.8	{11., 244.}	516.118	0.70994
45	newly 3 weeks spec	13.	10.61	{1., 22.}	57.3077	0.461227	newly 3 weeks cumul	46.	37.04	{8., 68.}	49.8261	0.367525	newly 5 weeks spec	7.	7.76	{0., 18.}	80.	0.533181	newly 5 weeks cumul	63.	53.74	{9., 114.}	53.7143	0.39209	newly 46 weeks cumul	86.	106.47	{9., 239.}	88.3605	0.483183
46	newly 3 weeks spec	11.	19.88	{6., 34.}	103.455	0.338592	newly 3 weeks cumul	54.	65.13	{21., 110.}	52.3148	0.230952	newly 5 weeks spec	9.	15.19	{1., 32.}	109.444	0.466674	newly 5 weeks cumul	77.	97.89	{27., 167.}	60.1948	0.262645	newly 53 weeks cumul	299.	196.01	{27., 419.}	50.8395	0.417737
47	newly 3 weeks spec	14.	15.56	{2., 31.}	62.4286	0.401589	newly 3 weeks cumul	32.	53.17	{10., 101.}	93.2813	0.320712	newly 5 weeks spec	10.	11.49	{0., 23.}	75.5	0.518048	newly 5 weeks cumul	50.	78.97	{11., 152.}	89.26	0.33259	newly 51 weeks cumul	260.	160.8	{13., 306.}	49.7	0.454213
48	newly 3 weeks spec	9.	9.5	{0., 21.}	77.1111	0.527337	newly 3 weeks cumul	23.	33.9	{5., 72.}	96.3478	0.418825	newly 5 weeks spec	3.	7.81	{0., 18.}	210.333	0.637555	newly 5 weeks cumul	34.	51.11	{5., 109.}	101.441	0.449435	newly 46 weeks cumul	83.	98.72	{5., 225.}	92.506	0.556156
49	newly 3 weeks spec	8.	13.37	{0., 31.}	121.125	0.561872	newly 3 weeks cumul	37.	46.31	{3., 106.}	79.7027	0.430589	newly 5 weeks spec	3.	9.94	{0., 26.}	281.333	0.720055	newly 5 weeks cumul	43.	67.01	{4., 162.}	105.093	0.472123	newly 50 weeks cumul	190.	131.92	{4., 313.}	60.6	0.624662
50	newly 3 weeks spec	19.	9.23	{0., 18.}	56.4737	0.629775	newly 3 weeks cumul	60.	31.74	{3., 64.}	54.5667	0.577617	newly 5 weeks spec	13.	6.9	{0., 17.}	62.1538	0.746151	newly 5 weeks cumul	91.	46.7	{3., 90.}	55.3626	0.615446	newly 47 weeks cumul	148.	91.83	{3., 187.}	59.3041	0.666621
